# The Heidelberg cardio-oncology unit (COUNT)—a possible blueprint for improved care of cardio-oncological patients

**DOI:** 10.1007/s00392-021-01894-z

**Published:** 2021-06-28

**Authors:** L. H. Lehmann, F. Stein, D. Jäger, N. Frey

**Affiliations:** 1 Department of Internal Medicine III: Cardiology, Angiology & Pulmonology, Heidelberg University Hospital, Heidelberg, Germany; 2 Cardio-Oncology Unit, Department of Internal Medicine III: Cardiology, Angiology & Pulmonology, Heidelberg University Hospital, Im Neuenheimer Feld 410, 69120 Heidelberg, Germany; 3 German Centre for Cardiovascular Research (DZHK), Partner Site Heidelberg/Mannheim, Heidelberg, Germany; 4 Deutsches Krebsforschungszentrum (DKFZ), Heidelberg, Germany; 5 German Cancer Consortium (DKTK), 69120 Heidelberg, Germany; 6 Department of Medical Oncology, National Center for Tumor Diseases (NCT), University Hospital Heidelberg, 69120 Heidelberg, Germany

Sirs:

As treatment has improved over the last decades, the long-term survival of patients with cancer, formerly associated with poor prognosis, has improved significantly. Cardiovascular diseases are common due to shared risk factors between cancer and heart disease. In many cases, cardiac comorbidities limit the optimal choice for oncological therapies. Additionally, cancer therapies themselves have unfavorable short- and long-term effects in cancer patients. Cardiotoxicity of targeted therapies is particularly important. With the expanding use of existing and the introduction of new targeted therapies, an increasing incidence of cardiovascular adverse events in cancer patients can be expected [1, 2]. Therefore, optimal and anticipatory management is imperative for optimal patient care. Conversely, even in advanced cancer, the cardiac disease contributes significantly to all-cause mortality and there is growing evidence that cardiological surveillance of cancer patients has the potential to improve quality of life and to reduce overall mortality in certain entities. [3] In cardiology, cancer patients turn out to be the high-risk population par excellence.

In 2010, the German Cancer Research Center and the ‘Deutsche Krebshilfe’ have established the first National Center for Tumor disease (NCT) in Heidelberg. This flagship-project served as a model to establish additional NCTs in Germany (Berlin, Cologne/Essen, Tübingen/Ulm/Stuttgart, Würzburg/Erlangen/Regensburg), as recently announced.

**Fig. 1:**
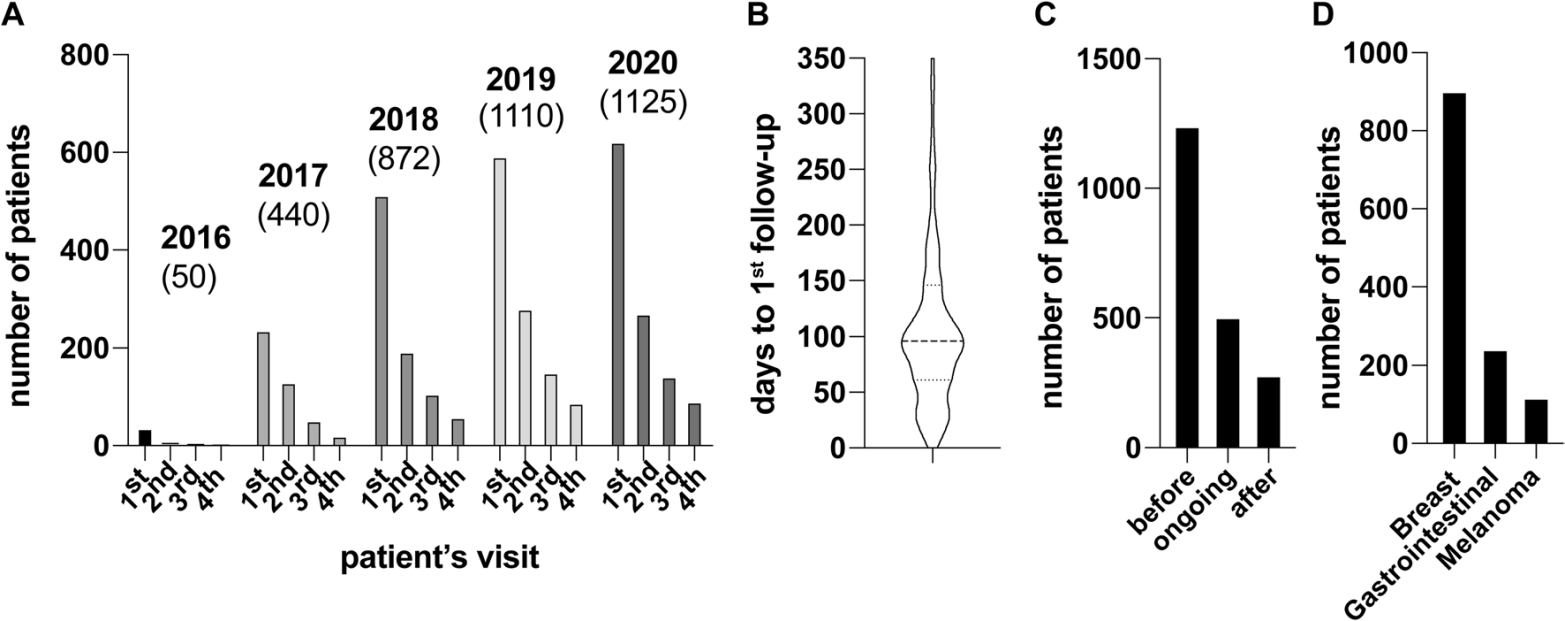
**A** Number of patients according to their visit as indicated with ordinary numbers. Shown are the years 2016–2021, numbers in brackets indicate a total number of visits in the COUNT. **B** Violin blot of the 1st follow-up visit. 43 follow-up visits were excluded (> 365 days after initial visit). **C** Number of patients before, during (ongoing) and after chemotherapy. **D** Predominant cancer entity of patients in the COUNT

In 2016, as an interdisciplinary project, the department of cardiology of the University of Heidelberg established the first German cardio-oncology Unit (COUNT), funded by the NCT 3.0 program and currently part of the NCT infrastructure in Heidelberg. Since then, the number of patients and visits has been growing continuously (Fig. 1A). The majority of patients were seen before starting initial or a new cancer therapy, and approximately 50% had at least one follow-up visit with a median of 96 days (Fig. 1A–C). At our COUNT, most patients suffered from breast cancer, followed by gastrointestinal cancer and malignant melanoma (Fig. 1D). Overall, more than 2000 patients were assessed with regard to echocardiographic parameters, cardiac biomarkers, ECG and—when available—by survival data. This is the result of an interdisciplinary follow-up approach for cancer patients in Heidelberg, aiming to track therapy success and to detect complications and adequately treat cardiovascular comorbidities.

The international awareness for this particular patient cohort is of growing interest and reflected by initiatives for COUNTs at other universities in Germany, such as Essen, Berlin, Cologne, Kiel and Hannover, and a number of clinical position papers that were published recently by the European Society of Cardiology (ESC) and the German Society of Cardiology (DGK) [4, 5].

From a cardiologist’s perspective—used to diagnose and treat patients with high levels of evidence—cardio-oncology suffers from the lack of sufficient prospective data. Especially randomized, multicenter trials are scarce. The diversification of cancer therapies and personalized strategies makes it even more challenging to set up prospective cardio-oncology trials, especially in cancer entities that are less frequent. Additionally, the underlying pharmacological mechanisms and associated cardiovascular events involve a broad range of possible molecular signaling pathways [6].

**Fig. 2:**
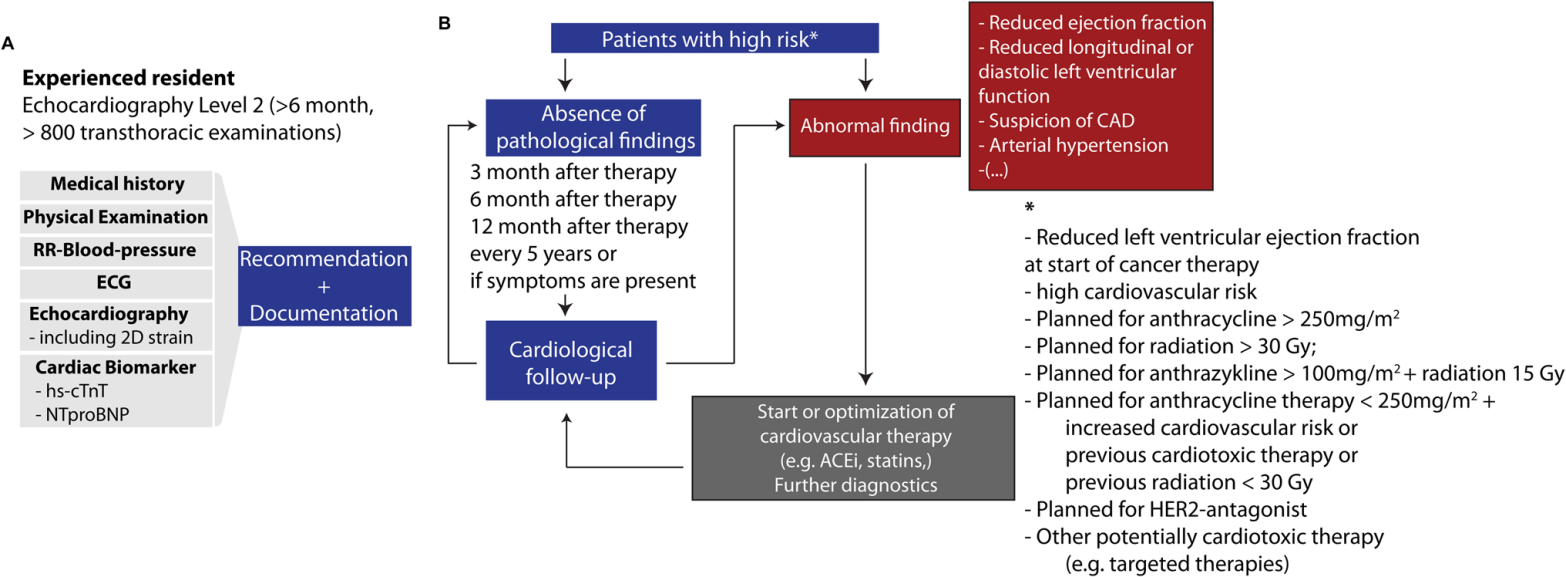
**A** Minimal list of examinations, needed for the cardio-oncology service. **B** Basic recommendation for the assessment of cancer patients in a COUNT (adapted from Lehmann et al. [10]). *hs-cTnT* high-sensitive cardiac troponin t, *ACEi* Angiotensin-converting-enzyme inhibitor, *ECG* electrocardiogram, *CAD* coronary artery disease

A standardized protocol consistent with the current recommendation of the DGK was implemented for the baseline (Fig. 2A) and surveillance examination (Fig. 2B) [5].

Timing of appointments in this ‘basic’ workup was adapted to certain patient cohorts. In case of suspected cardiotoxic events, patients undergo an early re-assessment and cardioprotective therapies are initialized. More specific cardiac imaging tools, such as cardiac MRI (cMRI) or specific PET-tracer were used in certain cases of suspected toxicity (checkpoint inhibitors associated myocarditis) and/or coronary artery disease [7].

For investigator-initiated trials, the COUNT serves as the cardiological backbone for studies evaluating cardiovascular adverse events associated with novel cancer therapies. Currently, the COUNT is part of 18 clinical cancer studies.

Own studies are focused on phenotyping of certain cohorts and integration of different data sets (among others, PET-CT scans, biomarkers and cMRI) [8]. At our unit, especially checkpoint inhibitor-associated myocarditis is a major focus of clinical research and part of international collaborations [9].

Based on clinical findings and phenotyping of patients, a number of preclinical projects are currently funded by the German Research Foundation (DFG) and the German Centre for Cardiovascular Research (DZHK). Clinical observations guide a translational (bed- to benchside) approach—with state-of-the-art in vivo and in vitro* models—*contributing to a better understanding of underlying pathologic molecular events.

Most importantly, the communication with the oncologist is crucial to achieve the best outcome for the patient. In the initial phase, the interaction between oncologists and cardiologists has intensified by participation in tumor boards and repeated presentations of cardio-oncological topics, but further referral and treatment strategies have to be developed and validated [10].

During patient care/examination, the entire documentation is done digitally, which includes cardiological findings and a brief summary of the recommendation from a ‘cardiologist’s perspective’. In case of severe findings that affect current or future oncological strategies, personal interaction is required.

In addition to research- and clinical- tasks, the topic of cardio-oncology was also integrated into the student’s curriculum of the NCT in Heidelberg which will further raise awareness of cardiovascular complications in cancer patients and could shape interdisciplinary career paths of future physicians.

The recent announcement of the integration of other partner sites as a nation-wide network of NCTs is a unique opportunity to further integrate cardio-oncological care as a new standard into strategies of cancer care in Germany, ideally also in all comprehensive cancer centers (CCC) in the future. Registries for cardiovascular side effects and a more personalized cardiological care will allow to answer important questions on how to predict, diagnose and treat cardiovascular pathologies in cancer patients. Structural integration into the NCT partner sites, standardization of decision making, the patient allocation for diagnosis and treatment of cardiotoxic side effects as well as optimal care for cardiovascular comorbidities currently performed at the COUNT, could serve as a template for a role out within the NCT and beyond.

By this, nationwide standardized cardio-oncological assessment in specialized COUNTs might contribute to patient wellbeing and further increase survival rates for cancer patients in general.
